# Predicting the Risk of Proteinuria Based on Blood Plasma Fourier‐Transform Infrared Spectroscopy and Machine Learning Algorithms From Pregnant Women and Newborns

**DOI:** 10.1002/ansa.70110

**Published:** 2026-10-09

**Authors:** Camila Lopes Ferreira, Sara Maria Santos Dias da Silva, Luma Martins Aleixo de Oliveira, Vitórya Carvalho Pádua de Magalhães, Simone Lima Silva, Sheila Cavalca Cortelli, Rodrigo Augusto Silva, Jaqueline Maria Brandão Rizzato, Marcelo Saito Nogueira, Luís Felipe Das Chagas e Silva de Carvalho

**Affiliations:** ^1^ Health Science Post‐Graduate Program University of Taubaté – UNITAU Taubaté Brazil; ^2^ Dentistry, University of Taubaté – UNITAU Taubaté Brazil; ^3^ Department of Electronic and Computer Engineering University of Limerick Limerick Ireland; ^4^ Health Research Institute (HRI) University of Limerick Limerick Ireland

**Keywords:** blood plasma, FTIR spectroscopy, machine learning, maternal and child health, pregnancy hypertension, prenatal diagnosis

## Abstract

Proteinuria, defined by an abnormally elevated concentration of proteins in the urine, serves as a critical biomarker for the early detection and diagnosis of preeclampsia. Elevated proteinuria levels adversely affect both maternal and foetal health, increasing the risk of preterm birth and low birth weight. This study focused on the identification of proteinuria in blood samples through Fourier‐transform infrared (FTIR) spectroscopy as a diagnostic tool for real‐time diagnosis. Blood plasma collected during birth was evaluated in four study groups: pregnant controls (*n* = 12), pregnant women with proteinuria (*n* = 13), newborn controls (*n* = 12), newborns with proteinuria (*n* = 14). First, attenuated total reflectance‐FTIR spectra of plasma samples were collected for subsequent spectral smoothing, vector normalisation and machine‐learning classification with various supervised machine learning algorithms, including several types of Support Vector Machine, K‐Nearest Neighbours and decision trees. We obtained (76.1 ± 3.1)% accuracy, (72.1 ± 4.8)% sensitivity and (80.4 ± 3.8)% specificity for pregnant classification, and (90.6 ± 1.5)% accuracy, (83.3 ± 2.4)% sensitivity and (98.9 ± 1.6)% specificity for newborn classification. The vibrational modes corresponding to the predominant biochemical components in blood plasma of pregnant women and newborns were listed. Sensitivity and specificity results demonstrated that FTIR can be a promising tool for early diagnosis and monitoring of individual proteinuria. Although the proposed spectroscopic approach demonstrated promising diagnostic performance, the findings should be interpreted in light of clinical conditions that could affect the biochemical profile. Further studies involving larger and more heterogeneous populations are required to validate the proposed methodology.

## 1 Introduction

Proteinuria is defined as the pathological presence of proteins in urine. Persistent proteinuria can indicate kidney damage or failure [1]. In normal and healthy populations, daily protein excretion levels in urine typically do not exceed 150 mg [2]. During pregnancy, however, there is an expected increase in protein excretion, which can rise to values equal to or greater than 300 mg per day. Proteinuria is an important clinical marker in the evaluation of preeclampsia but is not required for diagnosis, as preeclampsia may occur in the absence of proteinuria when new‐onset hypertension is accompanied by maternal organ dysfunction or uteroplacental dysfunction [3]. Proteinuria levels not only have adverse effects on pregnancy and maternal health, but also on child health, potentially leading to premature birth and low birth weight [4]. Both cardiovascular physiology and renal physiology are altered during pregnancy. Notable changes include increased blood flow and glomerular hyperfiltration. These changes are thought to contribute to elevated levels of proteinuria and a decrease in serum creatinine by up to 25%, which plays a crucial role in providing energy for muscle contraction [5].

Proteinuria reflects increased glomerular permeability, allowing albumin and other plasma proteins to pass into the urine [2]. Although severe or persistent proteinuria may eventually contribute to hypoalbuminemia, particularly in the setting of severe preeclampsia or nephrotic‐range proteinuria, serum albumin concentrations are not directly proportional to urinary albumin loss [1]. During pregnancy, serum albumin concentrations are also influenced by physiological plasma volume expansion and changes in albumin metabolism and therefore may not decrease proportionally to urinary albumin loss [2, 5]. For this reason, urinary albumin excretion and serum albumin concentration should not be considered interchangeable biomarkers [1, 2].

Currently, the 24‐h urine collection test is considered the reference standard for quantifying urinary protein excretion [5]. However, its accuracy may be compromised by incomplete urine collection [6], as it requires the collection of all urine excreted by the patient over a 24‐h period [7]. In clinical practice, spot urine samples, preferably first‐morning urine, are commonly used as an initial screening approach because of their practicality and rapid availability. The detection of proteinuria in a spot urine sample may represent an early warning sign and can prompt further quantitative assessment. Dipstick testing and protein‐to‐creatinine or albumin‐to‐creatinine ratios obtained from spot urine samples are widely used as first‐line screening methods. However, positive findings may require confirmation using quantitative laboratory methods because of variability in urine concentration and collection conditions [2, 8, 9, 10]. Although 24‐h urine collection provides a quantitative assessment, it is time‐consuming and may delay diagnosis, which is particularly relevant in pregnant women requiring prompt clinical evaluation [2].

It has limitations due to inadequate sample collection, inconvenience to patients, spillage, and other factors [8, 9]. The rate of inaccurate 24‐h urine collection is significantly higher during pregnancy compared to non‐pregnant states and may approach 50%. A cutoff of 150 mg for a 12‐h urine collection has shown high sensitivity (92%, 95% confidence interval [CI] 86%–96%) and specificity (99%, 95% CI 75%–100%) when compared with 24‐h urine collection [11]. Therefore, a real‐time method for early proteinuria detection is clinically needed.

Preeclampsia is a pregnancy‐specific multisystem disorder characterised by widespread endothelial dysfunction and systemic biochemical alterations. These alterations may affect the molecular composition of maternal blood plasma, including changes in proteins, lipids, carbohydrates, nucleic acids, and other biomolecular constituents [2]. Proteinuria, although reflecting impaired glomerular permeability, is an important clinical manifestation of preeclampsia and has traditionally been incorporated into its clinical assessment [5]. We propose performing blood analysis with Fourier‐transform Infrared (FTIR) spectroscopy as a tool for molecular diagnosis using fingerprint biological samples in real time. FTIR spectroscopy has been utilised to develop new alternative screening and diagnostic methods based on body fluids [12, 13]. This technique operates on the principle of molecular absorption of radiation, providing detailed information about the structure and composition of samples [14]. The fingerprint region of FTIR spectroscopy, typically between 600 and 1800 cm^−1^, contains molecular‐specific information crucial for clinical and medical applications. This region enables optical biopsy, facilitating the diagnosis of lesions and pathologies characterised by molecular‐specific biomarkers found in human tissues, cells, and secretions [15].

Spectroscopic techniques have long been employed for the analysis of biological tissues with highly sensitive methods for biochemical characterisation. Among these techniques, Raman spectroscopy and fluorescence spectroscopy have been extensively studied. While both methods provide valuable molecular insights, they present inherent limitations when compared to FTIR spectroscopy, particularly in the analysis of biological samples.

Raman spectroscopy, based on inelastic light scattering, provides high molecular specificity without sample preparation. However, its use in biological tissues is limited by fluorescence interference from endogenous fluorophores, weak Raman signals requiring long acquisitions and high laser power, and shallow penetration depth. These factors can obscure signals, cause photodamage, and hinder the analysis of thick or complex tissues [12, 15, 16, 17, 18, 19]. On the other hand, Fluorescence spectroscopy enables sensitive detection of specific biomolecules through their intrinsic fluorescence or the use of external fluorophores. However, its application to tissue analysis is limited by photobleaching, which causes signal loss during prolonged excitation, and tissue autofluorescence, which increases background noise and reduces spectral specificity. In addition, variations in experimental conditions, such as pH and temperature, can affect the measurements and reduce reproducibility [20, 21].

In contrast, FTIR spectroscopy is a powerful tool for biological tissue analysis because it provides detailed biochemical information without labelling or extensive sample preparation. By measuring infrared absorption by molecular bonds, it generates characteristic vibrational spectra that reflect tissue composition. Compared with Raman and fluorescence spectroscopy, FTIR is less susceptible to fluorescence interference. It also enables rapid, label‐free analysis and, when coupled with focal plane array detectors, spatially resolved chemical imaging for comprehensive biochemical mapping of tissue sections, supporting clinical and research applications. Overall, while Raman and fluorescence spectroscopy offer unique benefits in molecular characterisation, their limitations, such as fluorescence interference, photodamage, and experimental variability, make them less ideal for routine biological tissue analysis. FTIR spectroscopy overcomes these challenges by offering a highly reproducible and label‐free approach, making it a superior technique for biochemical tissue characterisation and potential clinical diagnostics.

Based on blood analysis, patient classification through FTIR spectroscopy coupled with machine learning (ML) algorithms can offer new insights for potentially developing a rapid analysis method for detecting proteinuria in pregnant women and newborns. This approach leverages the ability of FTIR to distinguish molecular compositions in biological samples, which could aid in diagnosing conditions such as proteinuria more efficiently and swiftly.

Despite the clinical relevance of proteinuria during pregnancy and the limitations of current diagnostic methods, no studies to date have explored the use of FTIR spectroscopy for detecting proteinuria using maternal or neonatal blood samples. With this in mind, we used FTIR spectroscopy to analyse blood plasma samples from pregnant women with proteinuria or not and their newborns. This technique assesses the vibrational transitions of chemical bonds after exposure to infrared light, allowing us to study molecular compositions and identify potential biomarkers associated with proteinuria in both maternal and neonatal samples. To the best of our knowledge, our study is the first to show proteinuria detection by using FTIR spectroscopy as a potential real‐time approach in preventive medicine for both maternal and child health.

## 2 Materials and Methods

### 2.1 Clinical Approval and Patient Information

Participants were recruited at Municipal University Hospital of Taubaté (HMUT) and enrolled in an observational study approved by the Research Ethics Committee of the University of Taubaté (approval number CAAE: 34126120.9.0000.5501). The study flowchart was presented in Figure 1. All procedures were performed in compliance with relevant laws and institutional guidelines. All participants signed an informed consent form and were over 18 years old. The inclusion criteria were: pregnant who had started prenatal care during the first half of pregnancy, gestational age (GA) ≤ 20 weeks, single pregnancy, non‐smokers, at least one of the risk factors for development of pre‐eclampsia (PE) which are obstetric history, nulliparity, primiparity, history of PE in a previous pregnancy; obesity (BMI ≥ 30 kg/m^2^), age ≥ 35 years; chronic arterial hypertension or without a risk factor present but with a diagnosis of PE at the time of peripartum. The definition of chronic arterial hypertension during pregnancy followed the criteria of the American College of Gynaecology and Obstetrics [22].

**FIGURE 1:**
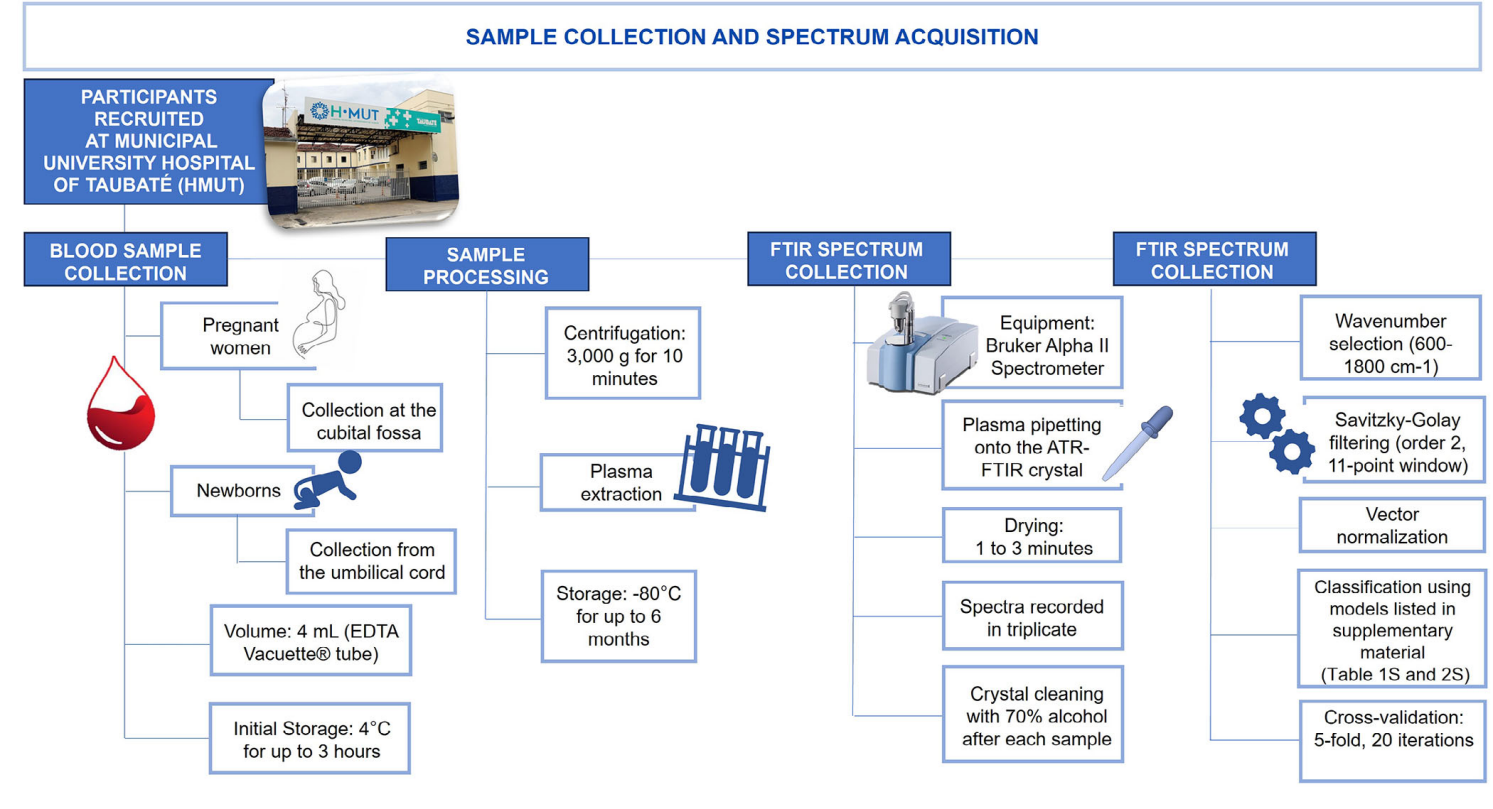
Study flowchart.

The exclusion criteria were: conditions that could interfere with the interpretation of the biochemical values of interest, pregnant women who did not reach the second half of pregnancy, gestational diabetes, autoimmune diseases and miscarriages.

### 2.2 Study Design

This is a cross‐sectional study, evaluating plasma samples at the moment of delivery from the following groups:

‐ Pregnant women (Control pregnant—CP); *n* = 12
‐ Pregnant women with Proteinuria (PP); *n* = 13
‐ Control Newborns (CN); *n* = 12
‐ Newborns with Proteinuria (NP); *n* = 14

### 2.3 Sample Collection and Processing

Pregnant and newborn blood collections were performed at HMUT. The venipuncture site for blood collection was the cubital fossa of pregnant women and the umbilical cord. The 4 mL tubes (EDTA Vacuette, Greiner Bio‐One Brasil) were identified previously, and then blood collection was stored at 4°C for up to 3 h, followed by plasma collection (VACUETTE Clot Activator Tube, Greiner Bio‐One Brasil). The sample was centrifuged at 3000 g for 10 min, and then the supernatant (plasma) was obtained and stored at ‐80°C for 6 months until FTIR processing.

### 2.4 Sample Processing and Data Collection

FTIR spectra of plasma samples were collected using a Bruker Alpha II spectrometer equipped with an attenuated total reflectance (ATR)‐FTIR diamond crystal and heating functions for sample drying, following a previous study [23]. These analyses were conducted at the Dental Research Centre (CEPEO‐UNITAU). For each FTIR measurement, 1 µL of plasma was deposited directly onto the ATR crystal without additives. The ATR crystal was maintained at 37°C using the instrument's heating function, and samples were subjected to a standardised drying period of approximately 1–3 min prior to spectral acquisition. The same temperature and drying procedure were consistently applied to all samples. Spectra for each sample were recorded in triplicate to ensure consistency and reliability. After each sample collection, the crystal was cleaned with 70% alcohol and allowed to dry to prevent cross‐contamination. Plasma samples were analysed within the FTIR spectral fingerprint region spanning 600 to 1800 cm^−1^, which provides detailed molecular information crucial for diagnostic purposes.

### 2.5 Data Analysis and Machine Learning

The spectral pre‐ and post‐processing procedures were executed using MATLAB (R2018a version; MathWorks, Natick, Massachusetts, USA) as described in Figure 2. The identification of the primary components in the samples was based on vibrational modes of the average FTIR spectrum of each study group and corresponding biochemical compounds as referenced in previous studies [24, 25]. Our spectral pre‐processing included smoothing the raw FTIR spectra using a Savitzky‐Golay filter with a second‐order polynomial and an 11‐point frame window, followed by Euclidean vector normalisation (2‐norm vector normalisation). The normalised FTIR spectra were subsequently utilised for plasma classification using various supervised ML algorithms, including Support Vector Machines (SVM). To maintain clarity and consistency in our results, we present only the performance metrics of the classifier that demonstrated the highest accuracy across all study groups, which were considered the most significant metrics for our feasibility study. The reported classification performance metrics are expressed as the mean and standard deviation, derived from 20 iterations of 5‐fold cross‐validation, with random sampling conducted for each iteration. Consequently, the mean values represent the average classification performance, while the standard deviations indicate the stability of the classification model with respect to the data distribution in our dataset. These preprocessed spectra (587 wavenumbers per sample) were used as input for supervised classification models. The entire dataset was randomly partitioned into a training set (80%) and a test set (20%), stratified by class labels to maintain group balance. This random partition was performed for every iteration of the 20 random samples in the 5‐fold cross‐validation. All ML models were trained exclusively on the training set and evaluated on the independent test set to prevent information leakage.

**FIGURE 2:**
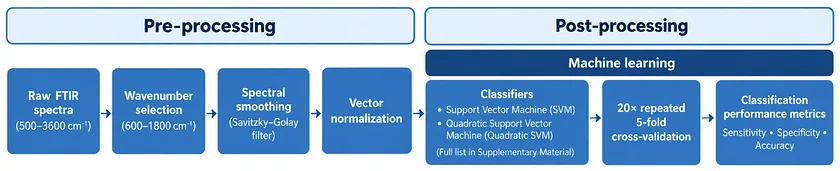
Spectral pre‐ and post‐processing procedures.

Data were randomised prior to partitioning into training and testing sets, and preprocessing steps were applied uniformly to all spectra. Class distributions were balanced using stratified sampling to prevent overrepresentation of any group. Additionally, analyses were performed in a blinded manner, ensuring that the data analyst was unaware of sample identities during model training and evaluation.

We implemented SVM, K‐Nearest Neighbours (with k = 5), and decision trees using MATLAB's Statistics and Machine Learning Toolbox. For SVM, a linear kernel was used due to its lower complexity and better interpretability in high‐dimensional spaces. The “box constraint” parameter (regularisation factor) was set to 1.0, and we performed additional sensitivity analyses by testing values between 0.1 and 10, which yielded consistent classification performance within a ±2% range. Five‐fold cross‐validation was employed within the training set to tune models and estimate generalisation error, and model robustness was confirmed through repeated stratified sampling (*n* = 5 iterations with different random seeds) [26, 27]. Multiple parameter combinations were tested to minimise the overall classification error while preventing model overfitting. The final parameters provided an optimal trade‐off between model flexibility and generalisation performance. This approach ensured that the classifier captured relevant spectral patterns without introducing excessive variance.

Performance was assessed using accuracy, precision, recall, F1‐score, and area under the receiver operating characteristic curve (AUC). To examine reproducibility, all classification steps were encapsulated in fixed‐seed routines and rerun across multiple independent folds, yielding standard deviations < 5% across metrics. These low deviations suggest minimal overfitting and confirm the pipeline's stability. All classification routines and analysis scripts are available upon request for independent verification. We emphasise that optimising feature selection for SVM classification [28] is not within the scope of this study, which focuses on the proof‐of‐concept proteinuria identification based on blood plasma FTIR spectroscopy.

To maintain clarity and consistency in our results, we present only the performance metrics of the classifier that demonstrated the highest accuracy across all study groups, which were considered the most significant metrics for our feasibility study. Metrics for other classifiers can be checked in Tables S1 and S2. Our reported classification performance metrics were expressed as the mean and standard deviation, derived from 20 iterations of 5‐fold cross‐validation, with random sampling conducted for each iteration.

For each fold of each iteration of 5‐fold cross‐validation, the dataset was separated into 80% for training and 20% for testing. Next, we trained the classification model with the training set. Then, the model was applied to classify and validate the test set. The process of model training and testing was repeated for all combinations of 80% training sets and 20% testing sets (i.e. the five combinations of 5‐fold cross‐validation). At the end of this process of one iteration of 5‐fold cross‐validation, the output classification performance metrics were the mean of each metric. The 20 iterations with random sampling ensure that the dataset is randomly shuffled for each iteration of 5‐fold cross‐validation. We reported the mean and standard deviation of the output of those 20 iterations. As a result, the mean values represent the average classification performance, while the standard deviations indicate the stability of the classification model with respect to the data distribution in our dataset.

The classification metrics calculated were based on parameters shown by confusion matrices defining the performance of each of our classification models based on the:

- True positive (TP): number of proteinuria patients correctly classified;
- False positive (FP): healthy control patients incorrectly classified;
- True negative (TN): healthy control patients correctly classified;
- False negative (FN): number of proteinuria patients incorrectly classified.

By using the above parameters for each iteration of 5‐fold cross‐validation, we calculated sensitivity, specificity and accuracy from:

$$Sensitivity=\frac{\mathrm{TP}}{\mathrm{TP}\;+\;\mathrm{FN}}$$

$$Specificity=\frac{\mathrm{TN}}{\mathrm{TN}\;+\;\mathrm{FP}}$$

$$Accuracy=\frac{\mathrm{TP}+\mathrm{TN}}{\mathrm{TP}\;+\mathrm{FN}+\mathrm{TN}+\;\mathrm{FP}}$$

and the area under the receiver operating characteristic (ROC) curve (from here on defined as AUC standing for “area under the curve”) was the integration over the ROC curve drawn by calculating the sensitivity (i.e., the true positive rate) against (1−specificity) known as the false positive rate at every possible classification threshold value.

It is worth noting that the clinical relevance of classification performance metrics in this study consists of the evaluation of the potential diagnostic potential of FTIR for proteinuria patient identification based on classification of blood plasma spectra. In this context, high sensitivity means few false negative results; hence, fewer proteinuria cases will be missed by FTIR. Similarly, high specificity means few false positive results, thus fewer healthy patients being mistaken as having proteinuria. Briefly, combined metrics such as accuracy and AUC indicate the overall performance of the classification (longer explanations are out of the scope of our study, as our paper is not a tutorial paper).

## 3 Results

Our study utilised machine‐learning techniques to classify spectra from pregnant plasma samples, with the goal of identifying the best method for daily clinical practice. The quadratic SVM was the optimal classification method for pregnant samples. Our results (Table 1) showed that quadratic SVM achieved (76.1 ± 3.1)% accuracy, (72.1 ± 4.8)% sensitivity, (80.4 ± 3.8)% specificity, and (79.6 ± 3.8)% AUC. These metrics indicate the effectiveness of quadratic SVM in accurately distinguishing and classifying spectra associated with pregnant plasma samples, demonstrating its potential utility in clinical settings. Figure 3 shows the average FTIR spectra of both pregnant groups, which were used.

**TABLE 1:** Pregnant confusion matrix.

Samples classified as	Control	Proteinuria
Control (*n* = 12)	(80.4 ± 3.8)%	(27.9 ± 4.8)%
Proteinuria *(n* = 13)	(19.6 ± 3.8)%	(72.1 ± 4.8)%

Legend: n = number of participants.

**FIGURE 3:**
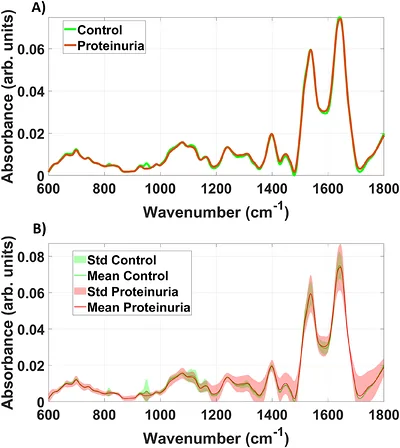
Fourier‐transform infrared (FTIR) spectra of pregnant groups. Legend: (A) Mean processed FTIR spectra of control pregnants (CP; green) and pregnants with proteinuria (PP; red); (B) Standard deviation (Std) of control pregnants (CP; green) and pregnants with proteinuria (PP; red).

For newborns, the best classifier was achieved with a linear SVM. Our results are shown in the confusion matrix of Table 2. We obtained (90.6 ± 1.5)% accuracy, (83.3 ± 2.4)% sensitivity, (98.9 ± 1.6)% specificity, and (95.9 ± 0.6)% area under the receiver operating characteristic curve (AUC). The spectra of newborns are shown in Figure 4.

**TABLE 2:** Newborn confusion matrix.

Samples classified as	Control	Proteinuria
Control (*n* = 12)	(98.9 ± 1.6)%	(16.7 ± 2.4)%
Proteinuria (*n* = 14)	(1.1 ± 1.6)%	(83.3 ± 2.4)%

Legend: *n* = number of participants.

**FIGURE 4:**
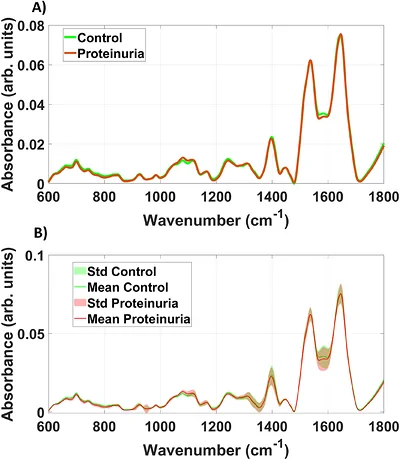
Fourier‐transform infrared (FTIR) spectra of newborn groups. Legend: (A) Mean processed FTIR spectra of control newborns (CN; green) and newborns with proteinuria (NP; red); (B) Standard deviation (Std) of control newborns (CN; green) and newborns with proteinuria (NP; red).

The bands and vibrational modes observed in all groups are summarised in Table 3 and Figure 5. Several vibrational modes were common across both pregnant and newborn groups, while others were specific to each condition. Specifically: The band at 816.3 cm^−1^, associated with ring CH deformation, was identified exclusively in the pregnant control group; A band at 950.5 cm^−1^, related to carotenoids and DNA, was also observed only in the pregnant control group and in newborns with proteinuria; and a band at 1307.0 cm^−1^, corresponding to the vibrational mode of amide III found in proteins, was identified. These spectral features highlight distinct biochemical compositions and molecular signatures associated with pregnancy and newborns under different health conditions, providing valuable insights into potential diagnostic biomarkers. Analysis of feature importance revealed that the spectral regions wavenumber bands at 816.3 and 950.5 cm^−1^ for pregnant controls and 1307.0 cm^−1^ for newborns with proteinuria potentially contributed most significantly to class separation. These regions correspond to molecular vibrations of carotenoids, DNA and proteins. The identification of these key bands enhances interpretability of the model and aligns with biochemical findings reported in related literature.

**TABLE 3:** Assignment of the main vibrational modes and biomolecular components to Fourier‐transform Infrared (FTIR) bands of each study group, following modes listed in [18, 19].

Bands (cm^−1^)	Vibrational modes	Biomolecular components	Groups
620.7	Unassigned	—	All
661.7	Unassigned	—	All
699.5	Out‐of‐plane bending vibrations		All
742.5	Unassigned	—	All
776.4	Unassigned	—	All
811.2	Ring CH deformation	—	All
816.3	Ring CH deformation	—	Pregnant (control)
831.7	C2' endo/anti (B‐form helix) conformation	Peptides and proteins	Newborn (control and proteinuria)
833.8	C2’ endo conformation of sugar, C2' endo/anti (B‐form helix) conformation, or Left‐handed helix DNA (Z form)	Peptides and proteins	Pregnant (control and proteinuria)
848.1	Left‐handed helix DNA (Z form) or C3' endo/anti (A‐form helix) conformation	Peptides and proteins	All
950.5	Symmetric stretching vibration of v1PO_4_ 3‐, or C deoxyribose, C‐C	Carotenoid	Pregnant (control)
984.3	OCH_3_	Polysaccharides–cellulose	All
1032.5	OCH_3_ stretching of methoxy groups, or V(C‐C) skeletal cis conformation, n(CH_2_OH), n(CO) stretching coupled with CO bending	Collagen	All
1044.8	C‐O‐H vibrations of the sugar moiety, OH stretching coupled with bending, C‐O stretching frequencies coupled with C‐O bending frequencies of the C‐OH groups	Carbohydrates (including glucose, fructose, glycogen, etc.)	All
1079.6	Symmetric phosphate [PO^2−^ (sym)] stretching, vs PO^2−^ or v PO^2−^	Collagen	All
1108.3	v(CO), v(CC), ring	Polysaccharides, pectin and cellulose	Newborn and Pregnant (proteinuria)
1161.5	CO stretching, stretching vibrations of hydrogen‐bonding COH groups, mainly from the CO stretching mode of COH groups, v(CC), d(COH), v(CO) stretching	Amino acids (particularly serine, threonine and tyrosine of proteins), peptides and proteins	Pregnant (control and proteinuria)
1167.7	C–O(H) stretching (Ser, Thr, and Tyr)	Amino acids (particularly serine, threonine and tyrosine of proteins), peptides and proteins	Newborn (control and proteinuria)
1239.4	Amide III, Symmetric and asymmetric PO^2−^ stretching, Collagen	Membrane phospholipids, collagen and other proteins	Pregnant (control and proteinuria)
1241.4	Amide III, Symmetric and asymmetric PO^2−^ stretching, Collagen	Membrane phospholipids, collagen and other proteins	Newborn (control and proteinuria)
1284.4	Amide III band, deformation N–H, N‐H thymine	Components of proteins	Pregnant (control and proteinuria)
1286.5	Amide III band, deformation N–H, N‐H thymine	Components of proteins	Newborn (control and proteinuria)
1307.0	Amide III	Proteins	Newborn (proteinuria)
1311.1	Amide III and CH_2_ stretching	Proteins	Pregnant (control and proteinuria)
1315.2	Amide III, CH_2_ stretching and vP=O	Lipid	Newborn (control and proteinuria)
1337.7	d(CH), ring (polysaccharides, cellulose), CH_2_ wagging and In‐plane CO stretching vibration combined with the ring stretch of phenyl	Collagen	All
1397.1	CH_3_ symmetric deformation	—	Newborn (control and proteinuria)
1399.2	Amide groups, CH_3_ symmetric deformation and bending modes, CH_3_ and CH_2_ vibrations, symmetric stretching vibration of COO‐ group, dsCH_3_	Proteins and lipids	Pregnant (control and proteinuria)
1442.2	d(CH_2_), d(CH)	Lipids, fatty acids, polysaccharides and pectin	All
1449.3	Asymmetric CH_3_ bending of methyl groups	Proteins	All
1468.8	dCH_2_, CH_2_ scissoring, and CH_2_ bending vibration	Lipids and proteins	All
1517 (shoulder)	Amide II	Peptides and proteins	All
1536.4	Amide II, Cytosine, Stretching C=N, C=C	Peptides and proteins	Newborn (control and proteinuria)
1538.5	Amide II, Cytosine, Stretching C=N, C=C	Peptides and proteins	Pregnant (control and proteinuria)
1571.2	Amide II, C=N adenine	Peptides and proteins	Pregnant (control and proteinuria)
1583.5	Ring C‐C stretch of phenyl	Lipids and proteins	Newborn (control and proteinuria)
1645.0	Amide I, C5 methylated cytosine, C=O, stretching C‐C uracil, NH2 guanine, dHOH	Peptides and proteins	All
1743.31 (shoulder)	C=O stretching, vC=O, ester CHO stretch	Lipids, triglycerides and cholesterol esters	Pregnant (control and proteinuria)
1747.4 (shoulder)	Ester group (C=O), v(C=O), v(C=C)	Triglycerides, polysaccharides, pectin, lipids and fatty acids	Newborn (control and proteinuria)

Legend: Bands indicated as “shoulder” indicate shoulder bands consisting of less perceivable peaks when a low‐absorbance peak appears over a broad FTIR peak with high amplitude (resulting in a small protuberance over the high‐amplitude peak, here referred to as “shoulder”).

**FIGURE 5:**
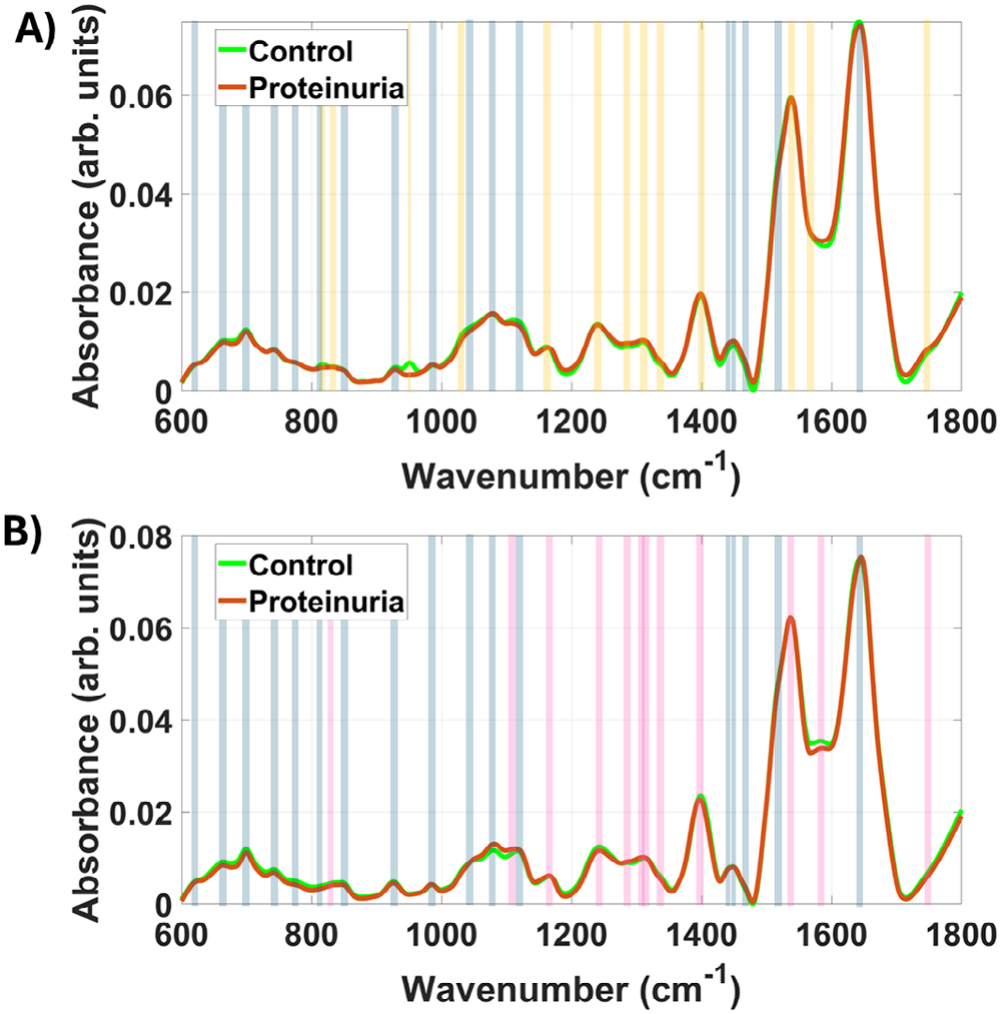
Fourier‐transform infrared (FTIR) spectral bands representing vibrational modes assigned to blood plasma of (A) pregnant women and (B) newborns. Legend: Bands marked in blue appear in plasma of both newborns and pregnant women. Bands marked in yellow appear only in plasma of pregnant women. Bands marked in magenta colour appear only in the plasma of newborns. Our assignment considered the predominant biochemical components in the mean spectra of each study group. For each group, the list of vibrational modes and corresponding biochemical components can be seen in Table 3.

## 4 Discussion

The present study investigated the blood plasma of pregnant women with proteinuria and their newborns using FTIR as a new diagnostic tool that utilises infrared light to generate spectroscopic data based on their molecular composition. By analysing FTIR spectral data, we found biomarkers present in plasma samples for diagnosing and understanding conditions such as proteinuria in both maternal and neonatal contexts. Timely diagnosis can potentially be used for clinical decision‐making prior to the 24‐h urine test, as FTIR can instantaneously show metabolic systemic changes occurring in newborns and pregnant women when combined with ML methods.

Given the large variability and inaccuracies expected from such urine tests, there is a need for alternative or complementary diagnostic approaches in clinical scenarios where diagnosis is required for fast decision‐making. Furthermore, the high variability of urine tests impairs not only the current evaluation of the impact of proteinuria on maternal health but also neonatal health (indirectly associated with maternal proteinuria and easier to predict based on plasma samples, according to our study).

The clinical need for faster, more convenient, cost‐effective and high‐throughput tests led to the development of reagent strip tests. Currently, these tests have automated readings that could potentially improve the protein estimation accuracy. However, this type of test lacks specificity and sensitivity due to its dependence on factors such as urine concentration (alkaline urine = false positive; acidic urine—false negative), the presence of pigments and dependence on the operator, requiring verification through a quantitative laboratory test [6].

A study aimed to compare the specificity and sensitivity results between visual and automated dipstick tests. Based on previous literature, a significant difference was noted between these two types of tests; however, the findings of this study concluded that the accuracy of these two methods may now be comparable. This variation in findings and results across studies could be a limiting factor for the use of these types of tests. In that study, only midstream urine samples were included, which may account for the differences in findings, highlighting that the method of urine collection directly impacts the results. Another factor noted was the rate of false positives and negatives, with the latter being particularly concerning, as they are not typically re‐evaluated, unlike false positives, which are further investigated through laboratory tests. Thus, both visual and automated dipstick tests show similar accuracy and are affected by the collection method, revealing a limitation in the diagnostic accuracy for proteinuria, demonstrating the need for more precise tests with fewer variables capable of affecting their results [10]. To compare available methods of proteinuria and our results, a table was added (Table 4).

**TABLE 4:** Performance characteristics of different proteinuria detection methods.

Method	Sample collection	Sensitivity (%)	Specificity (%)	Accuracy (%)	Precision (%)	AUC (IC 95%)
FTIR Spectroscopy (PP; our study)	Blood plasma sample	72.1 ± 4.8%	80.4 ± 3.8%	76.1 ± 3.1%	74%	—
FTIR Spectroscopy (NP; our study)	Blood plasma sample	83.3 ± 2.4%	98.9 ± 1.6%	90.6 ± 1.5%	85%	—
12‐h urine collection [29]	Urine sample	92% (95% Cl 75%–100%)	99% (95% CI 75%–100%)	—	—	—
Visual dipstick [10]	24‐h urine collection	0.69 (0.55–0.80)	0.70 (0.51–0.84)	—	—	—
Visual dipstick [10]	PCR (protein‐creatinine ratio)	0.68 (0.52–0.81)	0.92 (0.81–0.97)	—	—	—
Automated readers [10]	24‐h urine collection	0.65 (0.42–0.83)	0.82 (0.46–0.96)	—	—	—
Automated readers [10]	PCR (protein‐creatinine ratio)	0.73 (0.53–0.86)	0.91 (0.83–0.95)	—	—	—
Urine dipstick (PCR ≥0.2 g/g) [29]	Urine sample	90.9% (trace or more)	87.2%	—	—	0.935 (0.930–0.940)
Urine dipstick (ACR ≥300 mg/g) [29]	Urine sample	99.7% (trace or more)	82.6%	100%	—	0.983 (0.978–0.987)
Urine dipstick (ACR ≥30 mg/g) [29]	Urine sample	63.5% (trace or more)	93.5%	—	—	0.797 (0.783–0.812)

Legend: PP = Pregnant women with Proteinuria; NP: Newborn with Proteinuria; AUC = area under the receiver operating characteristic curve.

In our study, we obtained (72.1 ± 4.8)% sensitivity and (80.4 ± 3.8)% specificity for real‐time analysis of blood plasma samples with FTIR spectroscopy. Given that the sample analysis time is much shorter, FTIR spectroscopy can speed up clinical decision‐making compared with a cutoff of 150 mg for a 12‐h urine collection, with 92% sensitivity and 99% specificity [11]. Even with lower sensitivity and specificity, faster clinical interventions can improve both maternal and neonatal health, especially given that newborn blood plasma had even more pronounced FTIR‐sensitive biochemical changes than pregnant women—evidenced by higher classification performance metrics of FTIR newborn plasma classification such as (90.6 ± 1.5)% accuracy, (83.3 ± 2.4)% sensitivity, (98.9 ± 1.6)% specificity, and (95.9 ± 0.6)%. These biochemical changes can potentially be associated with the vibrational modes listed in Table 3. FTIR spectroscopy demonstrated enhanced diagnostic performance in neonatal samples, as per the results of our ML classifier based on our collected FTIR spectroscopic data. Enhanced classification performance in neonates is likely due to the simpler biochemical composition of neonatal matrices, which reduces spectral interference and improves the signal‐to‐noise ratio. In addition, neonatal samples often present fewer confounding metabolites, allowing clearer differentiation of characteristic absorption peaks.

A crucial point to consider is that the diagnostic performance of FTIR spectroscopy should not be interpreted as a direct measure of serum albumin concentration. Although albumin is the most abundant plasma protein and contributes substantially to the Amide I (approximately 1650 cm^−1^) and Amide II (approximately 1545 cm^−1^) spectral bands, these vibrational modes also receive contributions from other plasma proteins. Furthermore, the literature emphasises that the relationship between urinary albumin excretion and serum albumin concentration is non‐linear [1, 2]. Serum albumin often remains within the normal range during the early stages of proteinuria, as compensatory hepatic synthesis and physiological changes in plasma volume during pregnancy partially offset urinary protein losses; consequently, slight increases in urinary albumin are not necessarily accompanied by measurable decreases in circulating albumin. FTIR spectroscopy captures the global biochemical signature of plasma, integrating molecular changes involving proteins, lipids, carbohydrates, nucleic acids, inflammatory mediators and oxidative stress [13, 24, 25]. This comprehensive molecular profile supports the explanation of the proposed ML models’ ability to distinguish patients with proteinuria, even in situations where serum albumin concentration alone does not yet show significant alterations.

The association between proteinuria and the plasma FTIR profile may be explained by the systemic pathophysiological changes occurring in preeclampsia. Endothelial dysfunction and increased vascular permeability affect the glomerular filtration barrier, promoting urinary protein loss, particularly albumin. Concurrently, these processes may alter the concentration, distribution, conformation, and molecular environment of circulating plasma proteins. Such changes can influence protein‐related FTIR regions, especially the amide I and amide II bands, which are mainly associated with peptide backbone vibrations and protein secondary structure. In addition, the inflammatory and oxidative environment of preeclampsia may induce protein oxidation, aggregation, or conformational changes, while alterations in lipid and lipoprotein metabolism may affect lipid‐associated spectral regions. Therefore, the spectral differences observed in plasma are unlikely to represent urinary protein loss alone. Rather, they may reflect the broader systemic biochemical disturbances associated with endothelial injury, altered vascular permeability, inflammation, oxidative stress, and renal involvement. Since serum albumin was not measured in the present study, a direct relationship between urinary protein loss, circulating albumin concentration, and the observed spectral changes cannot be established.

To the best of our knowledge, there are no studies evaluating proteinuria samples through FTIR using plasma samples. Therefore, our research applied a new method to identify proteinuria in pregnant women and their newborns. This new analysis is FTIR, a spectroscopic analysis that can be implemented in either portable clinical instruments or high‐end microscopes for biochemical analysis, but it is also a versatile choice for diagnosis of multiple diseases in one test that can be combined with other clinical and laboratory exams [30].

In addition to the use of FTIR, another analytical method can be employed in cases of proteinuria, enhancing diagnostic accuracy. Generative Adversarial Networks (GANs) are an artificial intelligence technique that can be useful for generating images and distinguishing them as real or artificial. GANs could also be used to detect and analyse proteinuria in pregnant women and their newborns in further studies as a method of complementary imaging examination, using data produced by FTIR and ML, generating realistic images of higher levels of proteins in plasma samples [31]. It is essential to understand that the study of diagnostic techniques is always an attempt to assist clinicians with a new option, or rather, another option they can use to aid in diagnosis, and not necessarily that this technology will replace the existing one.

FTIR spectroscopy produces highly structured, multivariate data that naturally aligns with ML paradigms. Each FTIR spectrum comprises hundreds to thousands of absorbance measurements across discrete wavenumbers, yielding a high‐dimensional feature space ideal for pattern recognition tasks. This format is particularly suitable for supervised ML algorithms, which benefit from the consistent structure and low noise of FTIR spectral data. The ease of FTIR‐ML integration is also enhanced by the minimal sample preparation required. These features allow for rapid, reproducible acquisition of biochemical fingerprints from clinical specimens, such as saliva or other biofluids, which can be immediately subjected to ML‐based classification pipelines [32].

Moreover, FTIR is a technique capable of extracting intrinsic biochemical data through the vibrational energy of molecular bonds in the samples and of analysing all these components simultaneously, establishing itself as a tool that can assist in the early diagnosis of pathological conditions, using accurate and precise measurements of biological materials. Furthermore, it does not require sample preparation or high cost, and it enables automated analysis when integrated with ML. This integration between FTIR and ML makes it possible to create accurate computational simulations that can be easily used in the medical field. By associating FTIR with ML, it becomes possible to analyse large‐scale samples at low cost and, through portable devices, enable testing in treatment units and not only in laboratories [33].

FTIR provides a comprehensive and structured data matrix, while ML facilitates the interpretation of complex multivariate patterns that are often inaccessible through traditional analytical approaches. The synergy between FTIR and ML establishes a complementary framework that unites advanced analytical capabilities with predictive modelling, constituting an effective and reliable diagnostic methodology [32].

In terms of the origin and importance of proteinuria for maternal and neonatal health, it is important to note that the presence of excess protein in urine stems from two primary sources: glomerular barrier hyperpermeability and impaired proximal tubule protein reabsorption. There are substantially different characteristics between infants and adults, such as weight, height, blood volume, and levels of motor, mental, psychological, and histological development, making tumour progression different for each age group. In this way, normal protein levels in adults may be considered high in an infant, and high levels in infants may be minimal or normal in adults. Using this line of thought, the study by Mukherjee et al. employed a transfer learning mechanism to assist in the classification of pediatric brain tumours through prior classifications of adult tumours. In addition to the aforementioned differences, the heterogeneity and low volume of available data also authenticate the need for a method capable of aiding in the classification and diagnosis of pediatric tumours. Thus, Convolutional Neural Networks (CNNs) are utilised for these purposes. Similarly, as GANs can be allies in diagnosing proteinuria, CNNs can also be used in the diagnosis of this condition in newborns suspected of having proteinuria, facilitating and providing a more accurate diagnosis [34].

In addition to diagnostic accuracy, several practical and translational factors distinguish FTIR spectroscopy coupled with ML from other diagnostic modalities. One major advantage is cost‐effectiveness. FTIR instruments are generally less expensive and more economical to operate than other high‐throughput systems such as mass spectrometers or sequencing platforms. This makes FTIR‐ML diagnostics particularly attractive for large‐scale screening and resource‐limited settings. Portability and ease of implementation are also significant advantages of FTIR. Modern FTIR spectrometers are compact, robust, and capable of operating with minimal infrastructure, which facilitates their use in point‐of‐care environments. Moreover, because FTIR requires no chemical reagents or labels, sample processing time is significantly reduced, enabling rapid diagnostic workflows from collection to classification [32].

For example, if we compare it with HPLC associated with mass spectrometry, FTIR, in addition to being much cheaper, can, when associated with ML algorithms, obtain the result in real time. Finally, FTIR‐ML platforms offer high compatibility with clinical workflows, especially in the context of saliva‐based diagnostics. Unlike biopsies or blood draws, saliva collection is non‐invasive, well‐tolerated by patients, and ideal for repeated monitoring. When paired with ML algorithms, FTIR enables fast and accurate stratification of patient samples without requiring specialised personnel, thus opening avenues for decentralised, accessible diagnostic approaches.

The promising diagnostic performance observed in this study should be interpreted within the context of the study population, which was defined according to specific inclusion and exclusion criteria to minimise potential confounding factors affecting the biochemical profile. Consequently, the proposed classification models should be validated in larger and more heterogeneous cohorts, including women with different clinical characteristics and comorbidities. Such investigations, particularly through multicenter studies, will be essential to evaluate the robustness, reproducibility, and clinical applicability of the identified spectroscopic biomarkers across a diverse population. Serum albumin levels were not assessed, preventing the evaluation of the relationship between urinary albumin loss and circulating albumin concentrations. Future studies should simultaneously measure urinary and serum albumin levels to provide a more comprehensive understanding of albumin changes in preeclampsia.

It is important to highlight that pregnancy with proteinuria may develop pre‐eclampsia during gestation but is not a mandatory condition for diagnosis and classification. Nevertheless, it is known that the manifestation of preeclampsia is widely perceived to be centred on hypertension and proteinuria, but clinical presentations could be variable in essence [35]. One study investigated some haematological parameters of mothers with preeclampsia and umbilical cord blood, demonstrating a strong association between preeclampsia and low birth weight, premature caesarean delivery and proteinuria [36].

## 5 Study Limitations

Limitations of our study include the absence of urine analysis using FTIR spectroscopy, the variability in sample types (pregnant women and newborns), and the lack of previous studies that have analysed similar results in blood using the same method. These factors should be considered when discussing our findings. Still, we emphasise that the lack of FTIR urine sample analysis did not impact our results, as such analysis is not necessary for data interpretation from our ML classification based on blood plasma. Urine samples would allow deeper analysis into the urine protein levels. Our plasma samples were sufficient to detect high levels of proteins in pregnant women and their babies, ensuring an accurate diagnosis.

In order to diagnose more quickly and accurately, further studies are necessary, including comparisons between urine samples and blood samples, both analysed using FTIR and ML to enable new results and standards. In addition, utilising more AI techniques, such as GANs and CNNs, as previously cited, can customise the analysis of each case and provide greater accuracy in the required diagnosis and treatment.

We emphasise that our study was designed for proof‐of‐concept classification of proteinuria patients by training and testing our ML models on a computer coupled with the FTIR spectroscopy instrument. To translate FTIR spectroscopy to the clinic, a portable FTIR spectroscopy instrument should seamlessly integrate instrument control and ML, automating spectral classification upon sample placement in the ATR‐FTIR crystal and the click of a button by the clinician. We aim to achieve this scenario in our future studies, potentially embedding our classification model into miniaturised computers, microcontrollers and low‐power portable device components such as field‐programmable gate arrays (FPGAs), as such automation is outside the scope of this study.

It is worth noting that our study was the first to evaluate proteinuria classification by using FTIR spectroscopy for a potential real‐time approach in preventive medicine for both maternal and child health, to the best of our knowledge. As with most first proof‐of‐principle studies, the sample size is limited and common among many FTIR studies [15, 17, 19, 23, 24, 32, 33, 37, 38, 39, 40]. Also, as pointed out by Su and Lee [13], “although some studies may be preliminary and the sample size may be limited, these investigations [reviewed in their study] demonstrated the strong potential of FTIR spectroscopy as a highly sensitive, specific and accurate cancer diagnostic tool that is valuable for further investigation and development”.

Furthermore, sample size calculation is difficult to carry out in studies using multivariate analysis with 587 variables such as ours. This is not only due to a lack of comparison to previous FTIR diagnostics research in proteinuria, but also the absence of statistical methods for power analysis for clinical studies using multiple variables, as current power analysis is based on the output of one variable or a few variables (e.g. how many patients should be recruited in a clinical trial investigating the significance of plasma protein concentration as the single study variable). Without appropriate power analysis methods available, sample size calculations are complex, and developing new power analysis methods is out of scope of our work. Still, we acknowledge that our sample size was small and a robust analysis would potentially require more patients based on estimates guided by the work of Beleites et al., who examined the performance of discriminant models as a function of the number of samples in FTIR spectroscopy [41, 42].

However, we emphasise that the results of our study are still valid despite relatively small sample sizes, which should not undermine the findings of our work.

Another consideration is that plasma was collected only after delivery (i.e., after birth), without prior analysis of blood samples using FTIR. Our study will be the motivator for future analysis of such blood samples to impact preventive medicine practices and the follow‐up care of newborns. With this in mind, early blood analysis using FTIR could potentially provide valuable insights into metabolic or biochemical markers for early detection and intervention strategies, as well as real‐time biochemical analysis by FTIR, which provides rapid results, with accuracy reaching up to around 80%. For instance, if a test indicates no proteinuria, it reliably confirms its absence. This accuracy was notably higher in newborns compared to pregnant individuals.

Finally, serum albumin was not measured; therefore, changes in serum albumin levels could not be quantified in patients with either less severe or more severe preeclampsia. Additionally, the relationship between serum albumin levels, urinary albumin loss, and disease severity was not investigated. Future studies should include paired measurements of serum and urinary albumin and stratify participants according to preeclampsia severity.

## 6 Conclusion

By classifying FTIR spectra obtained from plasma samples and identifying the biochemical signatures that discriminate controls from individuals with preeclampsia, we demonstrated that FTIR spectroscopy is a feasible approach for detecting proteinuria and for elucidating molecular alterations relevant to maternal and neonatal health. The method achieved classification accuracies of 80% for pregnant women and 83% for newborns. Considering the clinical demand for diagnostic tools that are both rapid and reliable, FTIR spectroscopy represents a promising analytical modality capable of supporting timely clinical decision‐making.

## Declaration of Generative Artificial Intelligence Use

In preparing this manuscript, artificial intelligence tools (specifically, ChatGPT by OpenAI) were used exclusively for language refinement, grammar checking, and improvement of text clarity. No Artificial Intelligence‐based tools were employed for building algorithms for data analysis, statistical evaluation, or generation of scientific content. All experimental design, data processing, and interpretation were performed by the authors.

## Author Contributions

**Camila Lopes Ferreira**: writing – original draft and writing – review and editing. **Sara Maria Santos Dias da Silva**: writing – review and editing, investigation and data curation. **Luma Martins Aleixo de Oliveira**: writing – original draft and writing – review and editing. **Vitórya Carvalho Pádua de Magalhães**: investigation and data curation. **Simone Lima Silva**: investigation. **Sheila Cavalca Cortelli**: methodology. **Rodrigo Augusto Silva**: methodology. **Jaqueline Maria Brandão Rizzato**: investigation and data curation. **Marcelo Saito Nogueira**: writing – original draft, writing – review and editing, software, formal analysis, data curation, supervision, validation, and visualisation.

## Conflicts of Interest

The authors declare no conflicts of interest.

## Supporting information




**Supporting File**: ansa70110‐sup‐0001‐Tables1S and 2S.docx.

## Data Availability

The authors have nothing to report.
