# Supplementary material for: Predicting the Risk of Proteinuria Based on Blood Plasma Fourier‐Transform Infrared Spectroscopy and Machine Learning Algorithms From Pregnant Women and Newborns
Source: Anal Sci Adv. 2026 Oct 9;7(2):e70110. doi: 10.1002/ansa.70110 (PMC13652351; doi:10.1002/ansa.70110)
Supplement: Supplementary file 1 [file ANSA-7-e70110-s001.docx]

**Supplementary material**

**Title: Proteinuria identification based on blood plasma FT-IR spectroscopy and machine learning algorithms from pregnant women and newborns**

**Table 1S:** Classification performance metrics for all models using vector normalized spectra to classify Control Pregnant women (CP) versus Pregnant women with Proteinuria (PP).

|  | **Classification performance metric** | | | | |
| --- | --- | --- | --- | --- | --- |
| **Classification model** | **Specificity** | **Sensitivity** | **Accuracy** | **AUROC_Control** | **AUROC_Proteinuria** |
| **Fine Tree** | 0.631 ± 0.086 | 0.610 ± 0.065 | 0.620 ± 0.045 | 0.647 ± 0.059 | 0.647 ± 0.059 |
| **Medium Tree** | 0.647 ± 0.093 | 0.629 ± 0.066 | 0.638 ± 0.046 | 0.668 ± 0.054 | 0.668 ± 0.054 |
| **Coarse Tree** | 0.653 ± 0.098 | 0.595 ± 0.091 | 0.623 ± 0.051 | 0.651 ± 0.059 | 0.651 ± 0.059 |
| **Linear SVM** | 0.870 ± 0.035 | 0.658 ± 0.035 | 0.761 ± 0.030 | 0.816 ± 0.016 | 0.816 ± 0.016 |
| **Quadratic SVM** | 0.804 ± 0.038 | 0.721 ± 0.048 | 0.761 ± 0.031 | 0.796 ± 0.038 | 0.796 ± 0.038 |
| **Cubic SVM** | 0.776 ± 0.053 | 0.726 ± 0.066 | 0.750 ± 0.039 | 0.776 ± 0.048 | 0.776 ± 0.048 |
| **Fine Gaussian SVM** | 0.512 ± 0.039 | 0.881 ± 0.033 | 0.701 ± 0.027 | 0.752 ± 0.037 | 0.752 ± 0.037 |
| **Medium Gaussian SVM** | 0.769 ± 0.042 | 0.688 ± 0.038 | 0.728 ± 0.029 | 0.790 ± 0.021 | 0.790 ± 0.021 |
| **Coarse Gaussian SVM** | 0.895 ± 0.048 | 0.309 ± 0.050 | 0.594 ± 0.021 | 0.693 ± 0.037 | 0.693 ± 0.037 |
| **Fine KNN** | 0.761 ± 0.045 | 0.726 ± 0.043 | 0.743 ± 0.030 | 0.743 ± 0.030 | 0.743 ± 0.030 |
| **Medium KNN** | 0.834 ± 0.039 | 0.459 ± 0.045 | 0.641 ± 0.030 | 0.720 ± 0.038 | 0.720 ± 0.038 |
| **Coarse KNN** | 0.000 ± 0.000 | 1.000 ± 0.000 | 0.513 ± 0.000 | 0.524 ± 0.000 | 0.527 ± 0.000 |
| **Cosine KNN** | 0.724 ± 0.044 | 0.632 ± 0.049 | 0.677 ± 0.029 | 0.728 ± 0.028 | 0.728 ± 0.028 |
| **Cubic KNN** | 0.822 ± 0.044 | 0.496 ± 0.037 | 0.655 ± 0.030 | 0.745 ± 0.034 | 0.745 ± 0.034 |
| **Weighted KNN** | 0.807 ± 0.039 | 0.586 ± 0.054 | 0.693 ± 0.034 | 0.775 ± 0.023 | 0.775 ± 0.023 |
| **Boosted Trees Emsemble** | 1.000 ± 0.000 | 0.000 ± 0.000 | 0.487 ± 0.000 | 1.000 ± 0.000 | 0.000 ± 0.000 |
| **Bagged Trees Emsemble** | 0.743 ± 0.066 | 0.714 ± 0.047 | 0.728 ± 0.040 | 0.772 ± 0.032 | 0.772 ± 0.032 |
| **RUSBoosted Trees Ensemble** | 0.800 ± 0.102 | 0.337 ± 0.141 | 0.563 ± 0.039 | 0.694 ± 0.092 | 0.391 ± 0.136 |
| **Subspace KNN Ensemble** | 0.708 ± 0.044 | 0.627 ± 0.065 | 0.666 ± 0.042 | 0.730 ± 0.043 | 0.730 ± 0.043 |

**Table 2S:** Classification performance metrics for all models using vector normalized spectra to classify Control Newborns (CN) versus Newborns with Proteinuria (NP).

|  | **Classification performance metric** | | | | |
| --- | --- | --- | --- | --- | --- |
| **Classification model** | **Specificity** | **Sensitivity** | **Accuracy** | **AUROC_Control** | **AUROC_Proteinuria** |
| **Fine Tree** | 0.757 ± 0.083 | 0.794 ± 0.059 | 0.777 ± 0.040 | 0.798 ± 0.041 | 0.798 ± 0.041 |
| **Medium Tree** | 0.766 ± 0.066 | 0.793 ± 0.051 | 0.780 ± 0.042 | 0.794 ± 0.043 | 0.794 ± 0.043 |
| **Coarse Tree** | 0.750 ± 0.056 | 0.788 ± 0.057 | 0.770 ± 0.038 | 0.796 ± 0.043 | 0.796 ± 0.043 |
| **Linear SVM** | 0.989 ± 0.016 | 0.833 ± 0.024 | 0.906 ± 0.015 | 0.959 ± 0.006 | 0.959 ± 0.006 |
| **Quadratic SVM** | 0.920 ± 0.027 | 0.840 ± 0.021 | 0.878 ± 0.016 | 0.919 ± 0.015 | 0.919 ± 0.015 |
| **Cubic SVM** | 0.886 ± 0.036 | 0.864 ± 0.032 | 0.875 ± 0.022 | 0.908 ± 0.022 | 0.908 ± 0.022 |
| **Fine Gaussian SVM** | 0.558 ± 0.048 | 0.965 ± 0.021 | 0.775 ± 0.023 | 0.878 ± 0.017 | 0.878 ± 0.017 |
| **Medium Gaussian SVM** | 0.862 ± 0.038 | 0.858 ± 0.031 | 0.860 ± 0.029 | 0.922 ± 0.016 | 0.922 ± 0.016 |
| **Coarse Gaussian SVM** | 0.851 ± 0.022 | 0.671 ± 0.030 | 0.756 ± 0.016 | 0.825 ± 0.009 | 0.825 ± 0.009 |
| **Fine KNN** | 0.853 ± 0.033 | 0.821 ± 0.030 | 0.836 ± 0.022 | 0.837 ± 0.022 | 0.837 ± 0.022 |
| **Medium KNN** | 0.722 ± 0.039 | 0.715 ± 0.039 | 0.718 ± 0.027 | 0.788 ± 0.018 | 0.788 ± 0.018 |
| **Coarse KNN** | 0.000 ± 0.000 | 1.000 ± 0.000 | 0.532 ± 0.000 | 0.474 ± 0.000 | 0.470 ± 0.000 |
| **Cosine KNN** | 0.719 ± 0.046 | 0.730 ± 0.035 | 0.725 ± 0.030 | 0.790 ± 0.023 | 0.790 ± 0.023 |
| **Cubic KNN** | 0.736 ± 0.053 | 0.715 ± 0.031 | 0.725 ± 0.028 | 0.789 ± 0.026 | 0.789 ± 0.026 |
| **Weighted KNN** | 0.836 ± 0.038 | 0.802 ± 0.030 | 0.818 ± 0.027 | 0.891 ± 0.028 | 0.891 ± 0.028 |
| **Boosted Trees Emsemble** | 1.000 ± 0.000 | 0.000 ± 0.000 | 0.468 ± 0.000 | 1.000 ± 0.000 | 0.000 ± 0.000 |
| **Bagged Trees Emsemble** | 0.816 ± 0.042 | 0.842 ± 0.036 | 0.830 ± 0.025 | 0.897 ± 0.016 | 0.897 ± 0.016 |
| **RUSBoosted Trees Ensemble** | 0.828 ± 0.092 | 0.485 ± 0.188 | 0.646 ± 0.065 | 0.724 ± 0.048 | 0.519 ± 0.168 |
| **Subspace KNN Ensemble** | 0.809 ± 0.044 | 0.826 ± 0.043 | 0.818 ± 0.034 | 0.898 ± 0.028 | 0.898 ± 0.028 |
